# Intestinal permeability correlated with chronic fatigue in a patient with long COVID—A case report and overview of the literature

**DOI:** 10.3389/fmed.2026.1725242

**Published:** 2026-02-02

**Authors:** Ingo Andus, Janine Büttner, Bettina Bochow-Fitzner, Frank Tacke, Christoph Jochum

**Affiliations:** Department of Hepatology and Gastroenterology, Charité – Universitätsmedizin Berlin, corporate member of Freie Universität Berlin and Humboldt Universität zu Berlin, Campus Charité Mitte and Campus Virchow Klinikum, Berlin, Germany

**Keywords:** chronic fatigue, gut permeability, intestinal permeability, long COVID, PASC, post-acute sequelae of COVID-19, probiotic treatment

## Abstract

**Background:**

Long COVID is a complex condition characterized by persistent symptoms such as chronic fatigue, cognitive impairment, and autonomic dysfunction. Emerging evidence suggests that the gut may play a role in the pathophysiology of long COVID, potentially contributing to systemic inflammation and symptom severity. Prior studies indicate intestinal permeability (IP) alteration in patients with long COVID. To date, there have been no reports on the assessment of intestinal permeability via carbohydrate absorption in individuals with long COVID.

**Case presentation:**

We present a 60-year-old female with long COVID who exhibited chronic fatigue and autonomic dysfunction for more than 3 years following SARS-CoV-2 infection. IP was assessed at five time points using a carbohydrate absorption test. Results revealed significantly elevated lactulose/mannitol (L/M) ratios during episodes of symptom exacerbation, including a second SARS-CoV-2 infection. Notably, clinically observed improvement in fatigue correlated with a reduction in IP. A probiotic regimen with *Bacillus coagulans* and *Bacillus subtilis*, combined with a second intervention using medicinal clay, led to further clinical improvement.

**Conclusions:**

This case report demonstrates a correlation between intestinal permeability alterations and long COVID symptom severity. Notably, to our knowledge, this is the first report assessing intestinal permeability in a long COVID patient using a carbohydrate absorption–based permeability test. It reinforces the emerging link between gut barrier integrity and long COVID pathophysiology and emphasizes the need for further studies to assess IP as a potential disease marker. Furthermore, the observed improvement with probiotic therapy highlights the need for further research into microbiome-targeted interventions for long COVID management. Larger studies are required to explore the mechanistic link between gut permeability and long COVID pathophysiology.

## 1 Introduction

Long COVID, also known as post-COVID-19 condition, post-COVID-19 syndrome, long-haul COVID, post-acute sequelae of COVID-19 (PASC), and chronic COVID syndrome, encompasses a variety of symptoms that persist for weeks or even months following initial recovery from a COVID-19 infection (1). The different terminology refers to what is commonly known as long COVID, with minor alterations regarding the exact definition by various national and international institutions and clinical guidelines. The World Health Organization (WHO) defines it as a chronic condition after a SARS-CoV-2 infection. It must be present for 3 months after infection, and the symptoms must be present for at least 2 months without any other explanation (2).

While most individuals with long COVID experience mild to moderate symptoms that resolve within a few weeks, some continue to endure debilitating effects that can significantly affect their quality of life (3). The relative risk of developing long COVID seems to increase according to the severity of the acute infection. However, looking at the prevalence, 90% of cases occur in patients with mild symptoms (4). The symptoms of long COVID can vary widely, but fatigue, breathlessness, cognitive difficulties (often referred to as “brain fog”), and joint pain are among the most commonly reported issues (5). The underlying mechanisms of long COVID are still under investigation; however, it is believed that factors such as immune dysregulation, inflammation, and potential organ damage may contribute to its persistence (6).

Although long COVID and Myalgic Encephalomyelitis (ME), Myalgic Encephalomyelitis/Chronic Fatigue Syndrome (ME/CFS) are considered distinct conditions, they exhibit significant overlaps in symptoms and potential pathomechanisms. Consequently, research into ME/CFS may provide valuable insights into understanding and treating long COVID. ME/CFS is a complex and debilitating condition characterized by an incompletely understood pathomechanism. The primary symptoms include persistent and significant fatigue, intolerance to physical activity, post-exertional malaise, cognitive impairments, and musculoskeletal or joint pain. Symptoms often exacerbate following physical or mental exertion (7). In the context of CFS/ME, it is hypothesized that altered gut permeability may occur, leading to increased intestinal inflammation and bacterial translocation. This process is believed to trigger neuroinflammation, potentially contributing to the onset or progression of ME/CFS (8–10).

Intestinal barrier function is finely regulated to balance protection against pathogens and facilitate the absorption of nutrients and fluids (6). Altered intestinal permeability (IP), often called “leaky gut,” suggests passage of harmful substances like toxins and bacteria through the intestinal epithelium. This condition contributes to systemic inflammation and immune responses, which have been implicated in various health issues, including chronic fatigue syndrome (9). While larger molecules can only pass the epithelium through the paracellular route, which is highly regulated by junctional complexes between the cells, smaller molecules can also pass along the transcellular route either via passive flux through membranes, aqueous pores, endocytosis, or even active carrier-mediated absorption (11).

Accumulating evidence indicates that a substantial proportion of individuals with long COVID exhibit gut microbiome alterations, characterized by reduced microbial diversity, depletion of short-chain fatty acid–producing taxa, and enrichment of opportunistic or pro-inflammatory microbes (12–14). Although several mechanisms have been proposed the mechanistic links between dysbiosis and long COVID symptoms remain poorly defined.

Despite growing evidence linking gut dysbiosis and barrier dysfunction to long COVID, intestinal permeability has so far not been directly assessed using carbohydrate absorption–based tests. In this case report, we present a patient with a prolonged history of long COVID alongside the results of intestinal permeability testing conducted at various time points. Our findings indicate a correlation between elevated intestinal permeability and the severity of fatigue symptoms. Additionally, interventional data on microbiome-targeted therapies remain scarce. This case report presents new clinical data on the effects of microbiome-targeted treatments, specifically probiotic intervention with Innovall FD™ (*Bacillus coagulans* and *Bacillus subtilis)* and medicinal clay therapy using Luvos-Heilerde imutox™.

## 2 Methods

The ethics committee of the Charité– Universitätsmedizin Berlin approved the study (EA2/245/18), and informed consent was obtained from the patient. Clinical data were collected retrospectively from the patient's medical records.

Intestinal permeability was assessed using a quadruple carbohydrate absorption test that included sucrose (20 g), lactulose (10 g), mannitol (5 g), and sucralose (2 g). Carbohydrates were administered orally after an overnight fast, and urine samples were collected at two intervals: 0–5 h (5 h fraction) and 5–26 h (21 h fraction). The recovery rates of each carbohydrate were analyzed using high-performance liquid chromatography, as previously described (15). The sucrose, mannitol, lactulose, and sucralose recovery rates were calculated as a percentage of the ingested dose. Recovery rates of the carbohydrates lactulose and mannitol were calculated as lactulose/mannitol ratio and used as the common established marker for intestinal permeability (IP). A control group's mean value +2 SD (standard deviation) defined the upper limit of normal IP (IP = 0.03) (16, 17). The carbohydrate sucrose was immediately digested by disaccharidases when it enters the small bowel and is therefore established as marker for the gastroduodenal permeability (17). Gastroduodenal and intestinal permeability were calculated from the 5-h fraction. Only sucralose is not metabolized by bacteria when it enters the colon and is therefore established as marker for colon permeability. Colon permeability was calculated from the 21-h fraction (18).

A group of 25 healthy volunteers of European origin without any signs of gastrointestinal disorders was included in the healthy control group (male/female (9/16), mean age 57 ± 13 years).

## 3 Case report

In this case, a 60-year-old woman who was formerly healthy developed symptoms of long COVID after having been infected with the SARS-CoV-2 virus in 2020 without hospitalization. SARS-CoV-2 infection was confirmed via RT-PCR. The patient had no relevant comorbidities (reported comorbidities: atopic dermatitis, intraductal papillary mucinous neoplasm, mild depression), was eating a normal Western diet, had a normal body weight (BMI 22.6 kg/m^2^), no family history of immunological disorders, no recent antibiotic use and no allergies. Regarding lifestyle factors, the patient reported no regular physical activity, full-time employment, smoking approximately six cigarettes per day since 2019, and low alcohol consumption limited to weekends. Long-term medication consisted of escitalopram 10 mg daily and acetylsalicylic acid (ASA) 500 mg as needed. The patient was administered the AstraZeneca COVID-19 vaccine (AZD1222) approximately 6 months before contracting SARS-CoV-2. Shortly after the initial infection, she recognized a strong need for rest breaks, reported limitations in her daily activities, dizziness, mental exhaustion, moderate fatigue, headaches, joint pain, cognitive impairments, and irritable bowel syndrome. Symptoms did not improve over the next month. An increased susceptibility to infections was also reported. In clinical assessment, the patient exhibited moderate fatigue, as indicated by a Chalder-Fatigue-Scale score of 23/33 points and a Bell CFIDS disability scale score of around 40–50. Furthermore, the COMPASS questionnaire revealed severe autonomic nervous system dysfunction, with a score of 51.52/100 points. To exclude other underlying diseases or organ involvement, abdominal MRI, sonography, gastroduodenoscopy, and cardiovascular and pulmonary examinations were performed without any clinically significant findings that could explain the symptoms. The physical examination was normal. No microbial infections were detected in stool samples.

**Figure 1:**
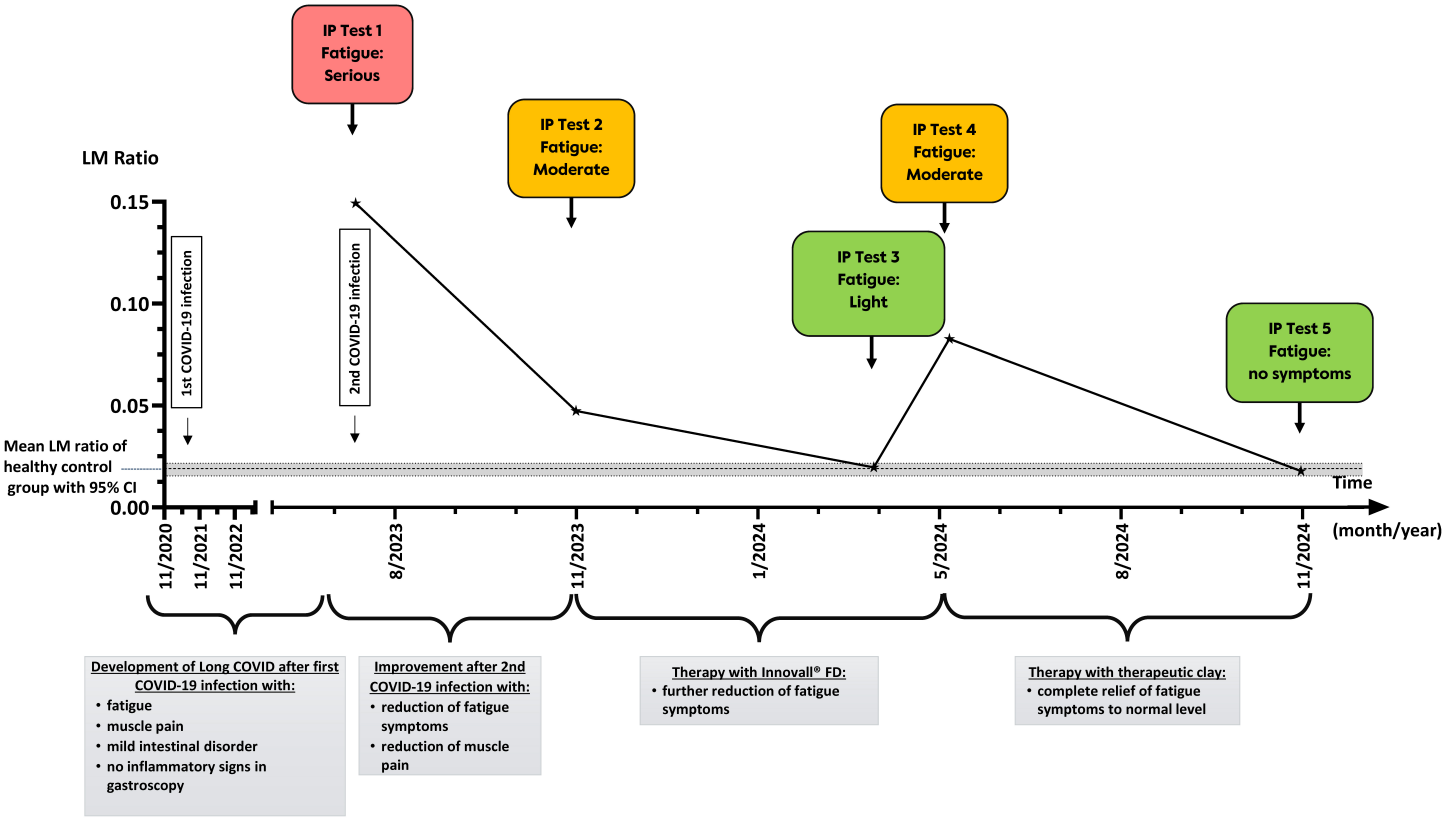
Analysis of lactulose/mannitol ratio in a patient with long COVID in relation to clinical findings and fatigue symptoms. IP testing (L/M ratio) was assessed at five different time points (IP Test 1–5). For each IP test, the intensity of fatigue symptoms was illustrated in colored squares (red: serious fatigue symptoms, yellow: moderate fatigue symptoms, green: light/no fatigue symptoms). Significant clinical findings and therapeutic interventions were added to the timeline for each interval between two IP tests. The dotted line indicates the mean L/M ratio of the healthy control group (*n* = 25), the gray bar represents the 95% confidence interval.

Twenty months after the first infection, the patient was examined at our gastroenterology outpatient clinic and consented to an analysis of intestinal permeability. By chance, the patient recognized her second SARS-CoV-2 infection during this intestinal permeability testing. The testing results showed significantly elevated lactulose and sucrose recovery rates, indicating a markedly increased paracellular permeability in the small intestine. In contrast, the recovery of mannitol (as a marker for transcellular permeability) and sucralose (as a marker for colonic permeability) remained unchanged compared to a healthy control group (*n* = 25). The calculated lactulose/mannitol ratio (IP-1, L/M ratio = 0.1493) for the patient was eight times higher than the mean ratio of the healthy control group (L/M ratio = 0.0186), the gastroduodenal permeability (sucrose recovery rate) was 15 times higher than the mean of the healthy control group (Figure 1 and Table 1).

**Table 1:** Recovery rates of carbohydrates in intestinal permeability testing.

**Carbohydrate recovery**	**5 h Sac (%)**	FC^*^(Sac%)	**5 h Man (%)**	FC^*^(Man%)	**5 h Lac (%)**	FC^*^(Lac%)	**21 h Suc (%)**	FC^*^(Suc%)	**Lac/Man ratio**	FC^*^(LM ratio)
IP-1	1.804	15.2	12.11	0.9	1.809	7.5	1.278	1.6	0.149	8
IP-2	0.422	3.5	10.15	0.8	0.481	2.0	0.588	0.7	0.047	2.6
IP-3	0.062	0.5	7.32	0.6	0.143	0.6	0.028	0.03	0.020	1.1
IP-4	0.352	3.0	4.91	0.4	0.405	1.7	1.143	1.4	0.083	4.4
IP-5	0.053	0.4	8.00	0.6	0.142	0.6	0.800	1.0	0.018	1
MEAN Controls (*n* = 25)	0.119		12.9		0.240		0.823		0.019	1
SD	0.091		4.29		0.072		0.702		0.006	
95% CI	0.07–0.12		11.4–13.8		0.21–0.26		0.66–0.89		0.015–0.022	

Results for carbohydrate recovery were given as a percentage of the ingested dose in urine collected for 5 or 21 h.

^*^Fold change (FC) is calculated as the estimated carbohydrate recovery in the patient divided by the mean carbohydrate recovery of the healthy control group (n = 25).

In the months following the second infection, the symptoms of long COVID, including fatigue, improved. Four months later, intestinal permeability testing showed reduced recovery rates for the paracellular markers sucrose and lactulose, compared to the assessment during active infection, but remained elevated compared to healthy controls. The calculated lactulose/mannitol ratio was more than twofold increased (IP-2), the gastroduodenal permeability showed a 3.5-fold increase (IP-2). Colon permeability was comparable to healthy controls. In the following, a sonography without any pathological findings was conducted.

The patient was then treated with Innovall FD™, a probiotic drug containing *Bacillus coagulans* MY01 and *Bacillus subtilis* MY02, administered at a dose of one capsule containing 2.5 × 10^9^ CFU (Colony Forming Units) twice daily for 3 months. During that time, her fatigue continued to subside, and the patient reported a reduction in symptoms, corresponding to an increase of around 20 on the Bell CFIDS disability scale from initial baseline of 40–60 [Scale is scored from 0 very severe−100 healthy (19)].

A third intestinal permeability test showed carbohydrate recovery rates comparable to the healthy control group (IP-3). Unfortunately, after the recovery period, the patient's symptoms worsened, and fatigue increased again (Bell CFIDS disability scale 40). A gastroduodenoscopy revealed only a very mild mucosal erythema. However, examination of mucosal biopsies revealed no pathological changes. A fourth intestinal permeability test showed increased recovery rates for sucrose and lactulose markers, indicating a re-increased paracellular permeability. The calculated lactulose/mannitol ratio (L/M ratio = 0.083) for the patient now was more than four times higher than the mean ratio of the healthy control group, and the sucrose recovery was three times increased, compared to the healthy controls. No changes were seen for the colon permeability (IP-4).

Following the relapse in clinical symptoms and increased IP, we explored an additional treatment method to the above-mentioned probiotic, using medicinal clay (mix of silicates, feldspar, smectite, calcite, dolomite, goethite) based on prior clinical experience at our center in patients with intestinal permeability disorders. We started a treatment using medicinal clay (Luvos-Heilerde imutox™) three times a day 3 × 900 mg capsules (total 8.1 g/day) of clay for 4 months. To prevent potential malabsorption of nutrients associated with medicinal clay, the treatment was then discontinued. Within 6 months following the initiation of medicinal clay therapy, the patient experienced a remission of symptoms (Bell CFIDS disability scale 100). Additionally, we saw a reduction in IP to normal levels and a normalization for all carbohydrate recovery rates (L/M ratio = 0.018, IP-5). In the clinical follow-ups, the patient remained in remission.

## 4 Discussion

### 4.1 SARS-CoV-2 infection and the gut

Infections with the SARS-CoV-2 virus primarily impact the respiratory tract; however, around 12% of patients also experience gastrointestinal symptoms, such as diarrhea, nausea, vomiting, anorexia, and abdominal pain (20, 21). The principal route of infection for SARS-CoV-2 is facilitated by binding of the viral spike protein to the ACE2 receptor on the surface of alveolar epithelial cells (22). Notably, the ACE2 receptor is also expressed on intestinal epithelial cells (23, 24). When comparing the relative expression, the ACE2 receptor is expressed 3 times more in the colon and 30 times more in the small intestine when compared to the expression in the lung (25). Furthermore, viral RNA has been detected in the stool of approximately half of COVID-19 patients, particularly in those exhibiting gastrointestinal symptoms (20, 26). Two main hypotheses exist to explain the entry of SARS-CoV-2 to the GI tract; one favors the route via oral ingestion (20), and the other favors an entry via the bloodstream (27).

### 4.2 Long COVID and the connection to the gut

Long COVID symptoms can include a wide range of ongoing symptoms and can affect several organs. Fatigue, brain fog, headache, and attention disorders are the most commonly reported symptoms, but 22% of patients also suffer from gastrointestinal symptoms like abdominal pain, nausea, dyspepsia, and irritable bowel syndrome (21). Notably, gastrointestinal symptoms are more commonly reported in long COVID (22%) than during the acute phase of SARS-CoV-2 infection (12%) (21).

The underlying mechanisms of long COVID are not fully understood. However, the evidence has identified several factors that are possibly involved. Suggested mechanisms include viral persistence, immune dysregulation, microbial translocation, coagulation disorders, neuroinflammation, autonomic dysfunction, and nerve damage to small fibers (1). The main GI-related mechanisms include viral persistence, gut dysbiosis, changes in the gut-brain axis, and intestinal permeability (10).

Diagnosis of long COVID in clinical routine is difficult, mainly because of the heterogeneity in symptoms and the lack of reliable biomarkers. A large multi-center study from the US with 10,094 participants investigated 25 routine clinical laboratory markers. It showed that routine clinical biomarkers could not distinguish between individuals with or without long COVID (28). The mechanistic differences in pathogenesis could guide a possible direction for further research (1). For example, a recent study by Swank et al. (29) investigated the mechanism of viral persistence, analyzing circulating viral antigens in 706 participants. A correlation between at least one long COVID symptom and detecting a positive antigen in blood samples was found in 43% of the participants. However, they could also detect circulating viral antigens in 21% of participants with no symptoms (29).

Gaebler et al. (30) also showed viral persistence in the gut by positive immunofluorescence staining of SARS-CoV-2 viral proteins in a subset of intestinal biopsies obtained from asymptomatic individuals 4 months after the onset of COVID-19. In a comparable manner, Zollner et al. (31) conducted an endoscopic intestinal biopsy study involving 46 patients with inflammatory bowel disease (IBD) approximately 7 months following mild acute COVID-19. The study demonstrated persistent viral nucleocapsid protein within the gut epithelium and CD8+ T cells in 52% of patients (24 out of 46) and detected SARS-CoV-2 RNA expression in 69% of patients (32 out of 46) (31). In line with these observations, Hany et al. prospectively analyzed endoscopic biopsies from patients with prior COVID-19 and identified persistent nucleocapsid protein by immunohistochemistry in 37.34% of upper gastrointestinal and 16.87% of lower gastrointestinal samples, based on 166 upper and 83 lower endoscopic procedures (32).

### 4.3 Intestinal permeability changes in long COVID patients

Studies investigating intestinal permeability during acute COVID-19 infection and in long COVID patients are currently limited. To our knowledge, results from carbohydrate absorption tests have not been reported in patients with active COVID-19 infection or those with long COVID. However, several studies have observed increased bacterial and fungal translocation, particularly in severe cases of COVID-19 (33–36).

A study by Yazici et al. (35) found significantly elevated levels of circulating bacterial DNA in the plasma of patients with severe COVID-19, suggesting an impaired intestinal barrier. This elevation was not observed in patients with mild COVID-19 cases (35).

Similarly, data from the EuCARE project assessed the lipopolysaccharide binding protein (LBP) levels during active infection and after viral clearance, showing that patients who developed long COVID had higher LBP levels during the acute infection compared to those patients who didn't establish long COVID afterwards (37). Increased LBP levels have been associated with intestinal barrier dysfunction. A preliminary study by Rohrhofer et al. (36), including a cohort of 30 long COVID patients, also found that these patients had higher serum LBP levels than convalescent SARS-CoV-2 participants and healthy controls (36).

Mouchati et al. (34) reported increased zonulin levels, suggesting increased IP in a large cohort of long COVID patients. In contrast, in their study, Giron et al. (33) did not observe a significant difference in zonulin levels, although observed absolute values were higher in the long COVID cohort. However, this variance might be caused by the unreliability of zonulin ELISA as a marker for intestinal permeability, as previously discussed in the literature (38, 39). These differences also highlight the need for further studies using more dependable methodologies to assess IP, such as carbohydrate absorption tests. Giron et al. (33) also observed an increase in fungal β-glucan levels in the blood, suggesting higher intestinal permeability in their group of long COVID patients compared to the control group.

Similar associations could be detected when looking at evidence from studies with ME/CFS patients (8, 9). For example, Maes et al. (8) measured bacterial translocation serving as a marker for increased IP by detecting IgA- and IgM-antibodies against bacterial lipopolysaccharides (LPS) in 41 ME/CFS patients and found a significant correlation with improvement of clinical symptoms in ME/CFS patients and a decrease in LPS-antibodies. Similar results were reported by Martín et al. (9), who compared LPS levels in 30 patients with ME/CFS and 26 matched healthy controls, revealing a positive correlation between clinical symptoms and increased LPS levels (*r* = 0.89; *P* < 0.001).

In this case report, we show a correlation between intestinal permeability (IP) and the severity of long COVID symptoms, suggesting that IP testing may serve not only as a potential disease marker but also as a tool to identify patients who could benefit from targeted therapeutic interventions addressing gut barrier dysfunction.

We present a 60-year-old woman suffering from long COVID for over 3 years with fatigue and severe autonomic nervous system dysfunction. Notably, the patient described only mild gastrointestinal symptoms with no diarrhea in this case. Further diagnostics showed a normal abdominal ultrasound, only some mild erythema in one of the endoscopies of the upper GI, and no pathological findings in the intestinal biopsy, even though the IP testing showed significantly increased IP. This could suggest that an increase in IP might not be limited to the long COVID subpopulation with GI symptoms. In our case, an observed eightfold and 4.4-fold increase of IP compared to a healthy control group correlated with the second active infection and an episode of relapse in long COVID fatigue, respectively. Conversely, a measured decrease in IP was associated with a clinically observed reduction in fatigue. Our results show an increase in paracellular permeability in the upper gut, correlating with the clinical severity of symptoms.

These findings further support that an impaired intestinal barrier may play a role in long COVID and chronic fatigue syndrome, as previously hypothesized (8, 9, 33, 34, 36, 37). Although more research with more participants is needed, our findings suggest IP may explain the clinical severity of long COVID symptoms and may even be a tool for diagnosing long COVID.

### 4.4 Gut dysbiosis and long COVID

Furthermore, current evidence suggests that gut dysbiosis is important in long COVID. For example, a meta-analysis from 2022 by Farsi et al., including 1,255 confirmed COVID-19 patients, showed a significant change in gut microbiome composition in COVID-19 patients (40). Liu et al. (12) conducted a prospective study examining gut microbiota composition in patients with post-acute COVID-19 syndrome (PACS) and identified significant microbial dysbiosis correlated with persistent symptoms. The study observed lower levels of short-chain fatty acid (SCFA)-producing bacteria, such as *Bifidobacterium pseudocatenulatum* and *Faecalibacterium prausnitzii*, in long COVID patients compared to controls (12). In a Brazilian cohort of 149 long COVID patients, 16S rRNA sequencing revealed gut microbiome shifts compared to healthy controls, marked by enrichment of dysbiosis-associated genera (*Desulfovibrio, Haemophillus, Dialister*, and *Prevotella*) and depletion of beneficial microbes such as *Bifidobacterium* and *Akkermansia* (41).

According to Dalile et al. (42) SCFA might influence gut–brain communication and brain function through immune, endocrine, vagal and other humoral pathways: SCFA have been proposed to influence intestinal barrier integrity and mucosal immunity through interactions with intestinal epithelial and immune cells, potentially involving activation of free fatty acid receptors, inhibition of histone deacetylases, modulation of tight junction protein expression and transepithelial electrical resistance. They have also been suggested to modulate systemic and neuroinflammatory responses, for example by influencing interleukin secretion or signaling via vagal afferents. Furthermore, SCFAs may affect central nervous system function through proposed effects on microglial activity, blood–brain barrier integrity, neurotrophic signaling, and neurotransmitter synthesis, thereby potentially influencing neural function, learning, memory, and mood (42). Microbial metabolites, such as short-chain fatty acids (SCFAs), are increasingly acknowledged as significant functional outputs of the microbiome that influence host physiology and immune function (43) and are currently discussed as one of the possible mechanistic links between gut dysbiosis and long COVID (13, 14, 44). Additionally, shifts toward pro-inflammatory microbial taxa and altered host–microbiome metabolic interactions may be involved in the development and persistence of long COVID (14, 45). Similar changes could be shown when looking at data for ME/CFS patients (46).

This indicates that gut microbiota might function as a marker and a therapeutic target for long COVID. Therefore, restoring microbial balance and intestinal barrier integrity, potentially through dietary interventions or probiotics, should be further investigated as a pathway for improving long COVID outcomes.

These findings prompted us to explore potential therapeutic approaches targeting intestinal permeability and gut microbiota as part of symptom management in long COVID. Given the observed correlation between improved intestinal barrier function and reduced fatigue in our case, we introduced a probiotic treatment regimen to evaluate its efficacy in modulating gut health. The patient in this report was given a probiotic (Innoval FD™) containing *Bacillus coagulans* and *Bacillus subtilis*. The patient reported reduced symptoms during the treatment, and the IP-test showed a concordant reduced permeability.

This compares to recent research underscoring the connection between COVID-19 and gut health. For example, comparable results could be found in the randomized, double-blind trial NCT04950803. The trial in Hong Kong assessed SIM01, a mixture of three bacterial strains (*B. adolescentis, Bifidobacterium bifidum*, and *Bifidobacterium longum*) with prebiotic compounds, in 463 participants with long COVID symptoms lasting at least 4 weeks. After 6 months, SIM01 significantly alleviated fatigue (63 vs. 43%), difficulty concentrating (62 vs. 39%), memory loss (42 vs. 27%), gastrointestinal upset (70 vs. 54%), and general unwellness (77 vs. 59%) compared to placebo. The benefits are attributed to SIM01's modulation of gut microbiota, improving diversity and promoting beneficial bacteria while reducing harmful species (47).

Another multicenter, randomized, double-blind, placebo-controlled clinical trial (Trial CTRI/2021/05/033576) in India tested a probiotic mixture *(Bacillus coagulans, Bacillus subtilis* and *Bacillus clausii*) in 200 patients with long COVID and fatigue symptoms for 14 days in a two-arm parallel design. The treatment efficacy was compared between the two groups using the Chalder Fatigue scale and showed a superior resolution of fatigue symptoms in the test arm compared to the placebo control arm (91 vs. 15%) at day 14 (48).

In summary, there is consensus that alterations of the gut microbiome are present in a substantial proportion of individuals with long COVID, based on data from multiple independent studies consistently reporting reduced microbial diversity, depletion of beneficial short-chain fatty acid–producing taxa, and enrichment of opportunistic or pro-inflammatory gut microbiota (12, 13, 47, 49). From a mechanistic standpoint, the available data remains incomplete, and no clear consensus has yet emerged. Several mechanisms have been proposed to explain how gut dysbiosis might contribute to long COVID, including impaired short-chain fatty acid metabolism (12, 50), altered tryptophan metabolism (14), increased microbial translocation due to barrier dysfunction (8, 9, 33, 34, 36, 37), resulting interactions with immune and inflammatory responses, and alterations to gut-brain axis signaling (12–14, 45). While these microbiome alterations seem to be a consistent feature of long COVID, the precise biological pathways linking dysbiosis to long COVID symptoms remain incompletely understood and warrant further investigation in longitudinal and mechanistic studies.

In line with these hypotheses, interventional approaches targeting the gut microbiome have shown potential symptom improvement (47, 48), as also observed in this case report. However, the available evidence is preliminary, heterogeneous, and insufficient to support definitive mechanistic or therapeutic conclusions. Despite therapeutic effects reported in multiple independent clinical studies, mechanistic causality has not been established, and it remains unclear to what extent dysbiosis represents a driver of long COVID vs. a consequence of behavioral changes, altered diet, reduced physical activity, or medication exposure during and after infection.

In addition to the probiotic intervention, a further therapeutic approach using medicinal clay was examined after the second relapse of symptoms with a concordant increase in IP (IP-4).

Research around medicinal clay is limited. However, existing data suggest that medicinal clays may act by adsorbing toxins, binding excess gastric acid, and alleviating symptoms of diarrhea and irritable bowel syndrome (51–53). Some studies also indicate an alteration in the microbiome (54–56), while others could not show any difference (57). Smectite, a principal component of medicinal clay, has demonstrated gastrointestinal cytoprotective effects in some studies. It is hypothesized to interact directly with the mucus layer by binding to mucins, modulating the composition and type of mucus secreted, and protecting the mucus barrier from bacterial mucolytic degradation, thereby strengthening its barrier function (58, 59). It has also been shown that smectite has the ability to absorb bacterial toxins such as *C. difficile* toxins, *C. perfringens* enterotoxin (60) and viral particles such as rotavirus (61). In this context, the adsorption of bacterial toxins and viral particles has been proposed as an alternative or complementary mechanism (61). Recent *in vitro* investigations utilizing Caco-2 cells demonstrated that diosmectide inhibited inflammatory responses in enterocytes by capturing SARS-CoV-2 particles, thereby obstructing subsequent inflammatory pathways and the interaction with ACE2 receptors (62, 63). One proposed mechanism for long COVID is viral persistence (1, 10, 30, 31). It may therefore be hypothesized that either the general antidiarrheal effects or the ability of medicinal clay to bind viral particles could contribute to the potential therapeutic benefits observed in this case report. While there is low-certainty evidence for the use of smectite in treating diarrhea in children (52, 64, 65) and adults (66, 67), further research is required. Without well-conducted multicenter randomized controlled trials and a clearer understanding of the underlying mechanisms, the benefits of using medicinal clay in long COVID remain unproven.

The patient reported reduced symptoms after administering medicinal clay in this single case report. The measured IP (IP-5) also showed normalization. In conclusion, we saw a correlation between the use of medicinal clay and remission of symptoms, further underlining a possible connection between the gut and long COVID pathophysiology, within the limitations outlined above.

These results suggest that microbiome-targeted therapies should be further investigated as potential effective interventions. It remains unclear to what extent increased intestinal permeability results from microbial dysbiosis or is an independent phenomenon that facilitates increased bacterial translocation.

### 4.5 Limitations

While these results highlight the growing evidence of the gut's critical role in long COVID pathophysiology (1, 10, 27, 29, 35), this is a single case report, and generalizations cannot be made from this report. Despite the limited data available, there is an urgent need for reliable diagnostic markers to better understand, diagnose, and manage long COVID. Our results further indicate that intestinal permeability and gut-related mechanisms could serve as a plausible marker for long COVID, as previously suggested in the literature (1, 9, 10, 25, 33, 34, 36). However, the mechanisms underlying increased intestinal permeability remain unclear, and further research is needed to confirm its role as a marker and to investigate the efficacy of probiotics and other microbiome-focused therapies. An additional limitation concerns the assessment of therapeutic effects of both interventions. Symptom severity was evaluated longitudinally during follow-up visits in our gastroenterology outpatient clinic and quantified as a patient-reported outcome using the Bell CFIDS Disability Scale. Given the complexity and heterogeneity of long COVID, future studies would likely benefit from the use of multiple scoring systems, to capture symptom burden more comprehensively. Moreover, as discussed above, the precise biological mechanisms underlying the observed effects of both interventions remain insufficiently characterized. Consequently, multicenter studies with larger patient cohorts, ideally placebo-controlled and randomized, as well as dedicated mechanistic investigations, are required to validate these findings.

## 5 Conclusion

This case report presents evidence of a correlation between intestinal permeability and the severity of fatigue in long COVID. To our knowledge, results from carbohydrate absorption tests have not been reported in patients with active COVID-19 infection or those with long COVID. We observed that elevated intestinal permeability in the upper gastrointestinal tract, measured by lactulose/mannitol ratio, coincided with increased fatigue, while improvement in gut barrier integrity matched clinical recovery.

Therefore, given the aforementioned limitations of this study, we hypothesize that intestinal permeability plays a role in the pathogenesis of long COVID. Testing for it may be a valuable tool in supporting the diagnosis of long COVID. However, further multicenter studies are necessary to validate the findings presented in this single case report.

Additionally, we could show promising results supporting the therapeutic potential of microbiome-targeted interventions in long COVID, aligning with emerging research, such as the Hong Kong SIM01 trial (47). With the introduction of a probiotic treatment containing *Bacillus coagulans* and *Bacillus subtilis*, we could show an improvement in clinical symptoms and intestinal barrier function. We also saw corresponding clinical improvements in a second intervention with medicinal clay, further supporting a clinically relevant connection between gut dysbiosis and long COVID. Both therapeutic interventions described in this case report represent potential treatment strategies that require further validation through larger, controlled trials.

## Data Availability

The original contributions presented in the study are included in the article/supplementary material, further inquiries can be directed to the corresponding author.
